# Identification of pivotal genes associated with the prognosis of gastric carcinoma through integrated analysis

**DOI:** 10.1042/BSR20203676

**Published:** 2021-04-14

**Authors:** Zhenchao Ma, Jianwei Xu, Lixin Ru, Weihua Zhu

**Affiliations:** 1 Department of Radiation Oncology, Affiliated Huzhou Hospital, Zhejiang University School of Medicine, Huzhou Central Hospital, Affiliated Central Hospital Huzhou University, Huzhou, China; 2 Department of Radiation Oncology, The Second Affiliated Hospital of Soochow University, Suzhou, China; 3 Department of Gastroenterology, Affiliated Huzhou Hospital, Zhejiang University School of Medicine, Huzhou Central Hospital, Affiliated Central Hospital Huzhou University, Huzhou, China

**Keywords:** Differential gene expression analysis, Gastric cancer, Protein-protein interaction network, Tumor-infiltrating immune cell, Weighted gene coexpression network analysis

## Abstract

**Purpose:** Detecting and diagnosing gastric cancer (GC) during its early period remains greatly difficult. Our analysis was performed to detect core genes correlated with GC and explore their prognostic values.

**Methods:** Microarray datasets from the Gene Expression Omnibus (GEO) (GSE54129) and The Cancer Genome Atlas (TCGA)-stomach adenocarcinoma (STAD) datasets were applied for common differentially co-expressed genes using differential gene expression analysis and Weighted Gene Co-expression Network Analysis (WGCNA). Functional enrichment analysis and protein–protein interaction (PPI) network analysis of differentially co-expressed genes were performed. We identified hub genes via the CytoHubba plugin. Prognostic values of hub genes were explored. Afterward, Gene Set Enrichment Analysis (GSEA) was used to analyze survival-related hub genes. Finally, the tumor-infiltrating immune cell (TIC) abundance profiles were estimated.

**Results:** Sixty common differentially co-expressed genes were found. Functional enrichment analysis implied that cell–cell junction organization and cell adhesion molecules were primarily enriched. Hub genes were identified using the degree, edge percolated component (EPC), maximal clique centrality (MCC), and maximum neighborhood component (MNC) algorithms, and serpin family E member 1 (SERPINE1) was highly associated with the prognosis of GC patients. Moreover, GSEA demonstrated that extracellular matrix (ECM) receptor interactions and pathways in cancers were correlated with SERPINE1 expression. CIBERSORT analysis of the proportion of TICs suggested that CD8^+^ T cell and T-cell regulation were negatively associated with SERPINE1 expression, showing that SERPINE1 may inhibit the immune-dominant status of the tumor microenvironment (TME) in GC.

**Conclusions:** Our analysis shows that SERPINE1 is closely correlated with the tumorigenesis and progression of GC. Furthermore, SERPINE1 acts as a candidate therapeutic target and prognostic biomarker of GC.

## Introduction

Gastric cancer (GC) is an aggressive solid tumor malignancy with approximately 27600 estimated new cases and 11010 estimated deaths in 2020, which causes a huge socioeconomic burden to patients and their families [1]. Many lifestyle factors have been researched, with behavioral factors such as tobacco use, alcohol consumption and *Helicobacter pylori* (HP) infection and are regarded as risk factors contributing to the development of GC [2]. Currently, the therapeutic strategies for GC include surgery, radiotherapy, neoadjuvant chemotherapy and immunotherapy, and the survival rate for patients with early GC is nearly 90% [3]. However, it remains very difficult to detect and diagnose this cancer during its early stage, leading to an apparent reduction in survival rates after diagnosis [4]. Therefore, it is extremely necessary to detect candidate diagnostic and prognostic indicators and therapeutic targets for GC patients.

With the rapid progress of genomic technology, gene expression profiles are usually used through bioinformatics methods to explore the underlying molecular mechanisms of tumors and find cancer-specific indicators [5]. As a significant algorithm, Weighted Gene Co-expression Network Analysis (WGCNA) is widely applied to further understand gene co-expression networks and gene functions [6]. Based on WGCNA, modules of highly correlated genes correlated with the traits of samples are found, providing valuable insights into predicting the potential function of co-expressed genes [7]. Moreover, differential gene expression analysis is commonly adopted to analyze transcriptomics datasets, and this is conducive to exploring the underlying biological and molecular mechanisms of tumors and detecting quantitative differences between the gene expression levels of experimental and control cohorts [8].

The two approaches mentioned above are adopted in our analysis for the better capability of discriminating highly related genes. First, we acquired gene expression profiles of GC from the Gene Expression Omnibus (GEO) and The Cancer Genome Atlas (TCGA) databases. Second, WGCNA and differential gene expression analyses were applied to detect common differentially co-expressed genes. Then, we conducted functional enrichment analysis, protein–protein interaction (PPI) analysis and survival analysis for potential biomarkers related to the occurrence, development and prognosis of GC. Subsequently, the relationship of survival-related hub genes with clinical data was explored. Next, we performed gene set enrichment analysis (GSEA) of survival-related hub genes. Finally, the tumor-infiltrating immune cell (TIC) abundance profiles were estimated using the TCGA-stomach adenocarcinoma (STAD) dataset.

## Materials and methods

**Figure 1:**
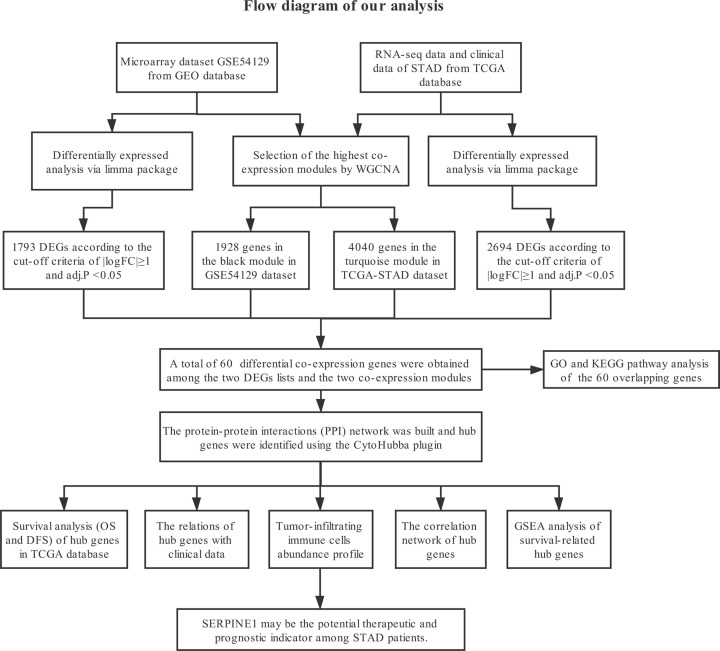
Study design and workflow of our study

The specific steps of how data were downloaded, hub genes were selected and TIC profiles of GC were estimated are illustrated in Figure 1. Each step is shown clearly in the subsequent subsections.

### Datasets download

We downloaded gene expression profiles of GC from the GEO and TCGA databases. GSE54129 was acquired from the GEO database and included 111 GC and 21 normal tissues. The GSE54129 platform was GPL570 [HG-U133_Plus_2] Affymetrix Human Genome U133 Plus 2.0 Array. Based on the annotation file provided by the manufacturer, probes would be transferred to gene symbols, probes without corresponding gene symbols would be removed, and average values would be taken when one gene corresponded to multiple probes. Consequently, we obtained 21655 genes for our next analysis. On the other hand, the gene expression dataset and clinical data of GC were acquired from the TCGA database (https://portal.gdc.cancer.gov/). A total of 407 samples from the TCGA were obtained, consisting of 375 GC and 32 normal tissues, as well as RNA-Seq count data of 19646 genes. Using the Illumina HiSeq 2000 platform, all of the data were generated and annotated to a reference transcript set of the human hg38 gene standard track. Moreover, the edgeR package tutorial revealed that genes with low read counts usually played an insignificant role in the next analysis [9]. Hence, we deleted genes with a count per million (CPM) < 1 from our study. Next, we applied the rpkm function in the edgeR package for further filtering. As a consequence, a total of 15085 genes were acquired for our next analysis.

### Identification of key co-expression modules using WGCNA

**Figure 2:**
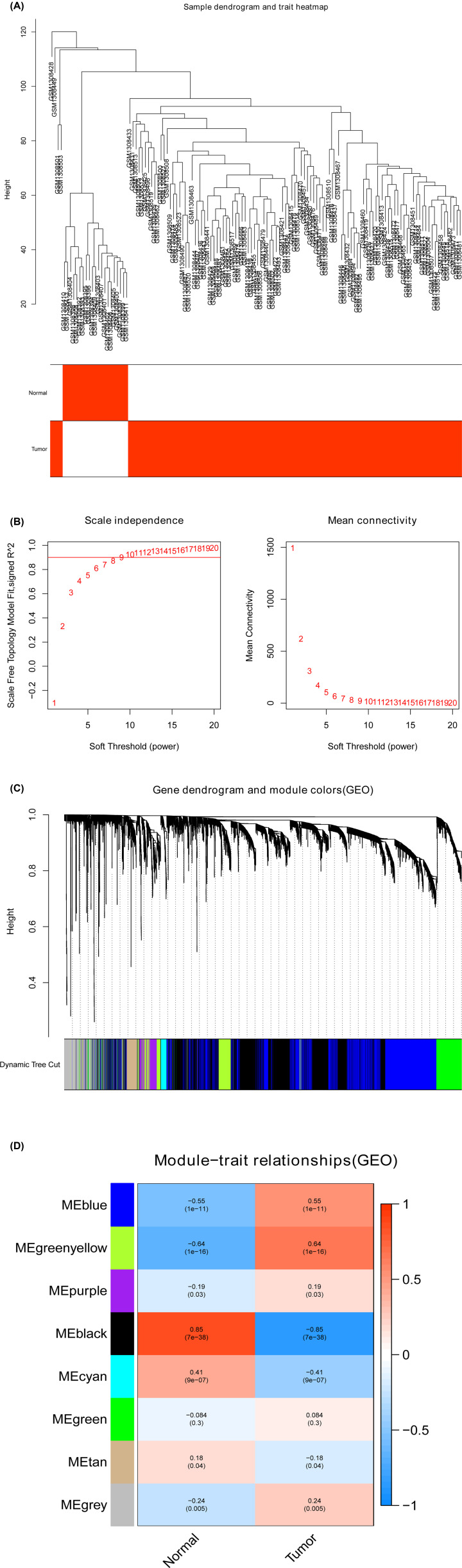
Identification of modules related to the clinical traits in GSE54129 (**A**) Sampl e dendrogram and trait heatmap. (**B**) Scale independence and Mean connectivity. (**C**) The Cluster dendrogram of co-expression network modules is ordered by a hierarchical clustering of genes based on the 1-TOM matrix. Different colors represent different modules. (**D**) Module–trait relationships. Each row represents a color module and every column represents a clinical trait (normal and tumor). Each cell contains the corresponding correlation and *P*-value.

**Figure 3:**
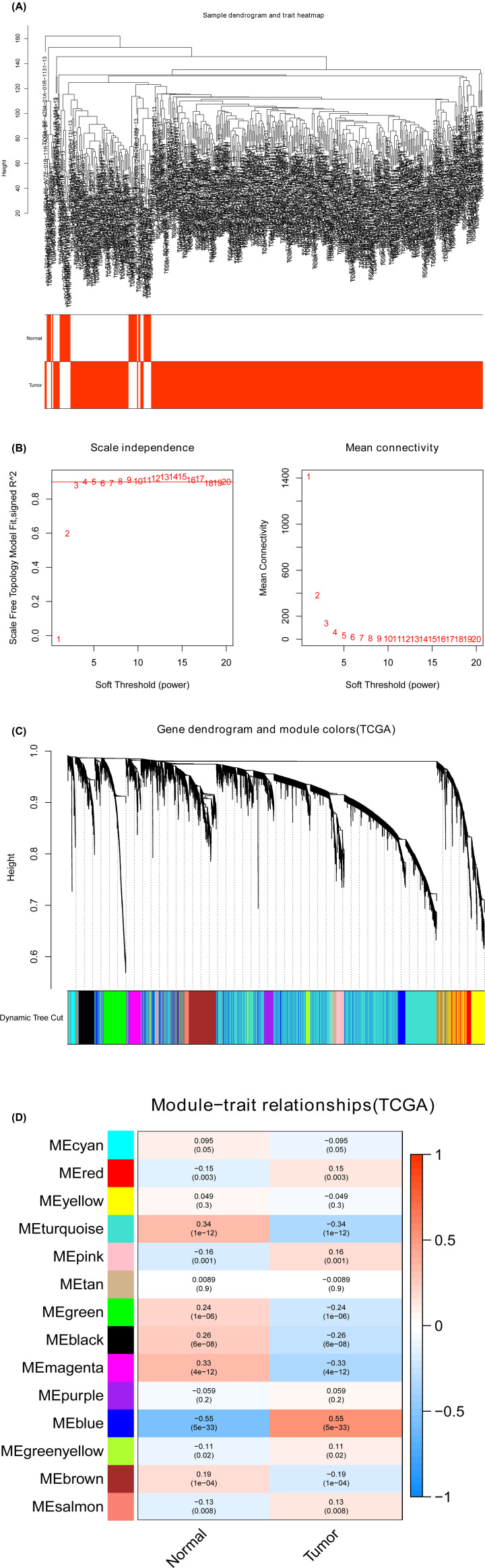
Identification of modules related to the clinical traits in the TCGA-STAD dataset (**A**) Sample dendrogram and trait heatmap. (**B**) Scale independence and Mean connectivity. (**C**) The Cluster dendrogram of co-expression network modules is ordered by a hierarchical clustering of genes based on the 1-TOM matrix. Different colors represent different modules. (**D**) Module–trait relationships. Each row represents a color module and every column represents a clinical trait (normal and tumor). Each cell contains the corresponding correlation and *P*-value.

The gene co-expression networks of GSE54129 and TCGA-STAD datasets were established via the WGCNA package [6]. WGCNA found highly related genes and aggregated these genes into the same co-expression module related to clinical traits. To establish the scale-free networks, soft-powers β = 8 (Figure 2A,B) and 3 (Figure 3A,B) were applied to the GSE54129 and TCGA-STAD datasets, respectively. Afterward, we created the adjacency matrix based on the following formula: aij = |Sij|^β^ (aij: adjacency matrix between gene i and gene j, Sij: similarity matrix which was made by Pearson’s correlation coefficient of all gene pairs, and β: soft-power value), and then this matrix was converted into topological overlap matrix (TOM) and the corresponding dissimilarity (1-TOM). The hierarchical clustering dendrogram of the 1-TOM matrix was built to aggregate genes with similar expressions into the same co-expression module. Subsequently, the module–trait relations between modules and clinical traits were explored for functional modules from the co-expression network. Thus, the module with the largest correlation coefficient was considered the potential module that was highly associated with clinical traits, and this module was used in the next analysis.

### Identification of differentially co-expressed genes

The limma package was usually adopted to conduct differential gene expression analyses of microarray and RNA-Seq datasets [10]. To detect differentially expressed genes (DEGs) between GC and non-tumorous tissues, the limma package was used in the differential expression analysis of the GSE54129 and TCGA-STAD datasets. To reduce the false discovery rate (FDR), the *P*-value was adjusted via the Benjamini–Hochberg (BH) approach. |logFC|>1 and adj. *P*<0.05 were used as the selection criteria for DEGs. We took the intersection of genes among two lists of DEGs and two lists of co-expressed genes for common genes to improve the capability of discriminating closely related genes, and the genes from this intersection were adopted for candidate biomarkers related to the prognosis of GC.

### Functional enrichment analyses

Gene ontology (GO) and Kyoto Encyclopedia of Genes and Genomes (KEGG) pathway analyses constituted functional enrichment analyses of the differentially co-expressed genes. To explore the biological functions of differentially co-expressed genes, GO and KEGG pathway analyses were conducted through the clusterProfiler [11] package. GO is an essential bioinformatics tool that is usually adopted to annotate genes and analyze their biological processes [12]. KEGG was conductive to understanding high-level functions and biological systems from large-scale molecular datasets [13]. *P*<0.05 was considered significantly different.

### PPI network construction and hub gene identification

The PPI network of these differentially co-expressed genes was established using the Search Tool for the Retrieval of Interacting Genes (STRING) [14]. Cytoscape was adopted to build the visual network of molecular interactions with a combined score >0.15 [15]. The Molecular Complex Detection (MCODE) plugin was applied to detect closely correlated modules from the PPI network [16]. The most significant gene module of this PPI network was visualized and displayed through the MCODE plug-in. The filtering criteria were as follows: MCODE score > 5, node score cutoff = 0.2, degree cutoff = 2, k-score = 2 and max depth = 100. Additionally, the degree, edge percolated component (EPC), maximal clique centrality algorithm (MCC), and maximum neighborhood component (MNC) algorithms were useful methods of selecting hub genes from PPI networks [17]. We calculated the degree, EPC, MCC and MNC scores of all nodes of the PPI network via the CytoHubba plugin. The top 10 nodes with the highest degree, EPC, MCC and MNC scores were selected, and we took the intersection of outcomes of the four algorithms. To increase the reliability of hub genes, their overlapping genes were believed to be hub genes related to GC.

### Survival analyses of hub genes

To screen the prognostic values of these hub genes, we conducted univariate Cox regression analysis of the overall survival (OS) via the survival package using the TCGA-STAD dataset. We also conducted disease-free survival (DFS) analysis of hub genes in The Gene Expression Profiling Interactive Analysis (GEPIA) database (http://gepia.cancer-pku.cn/) [18]. STAD patients with missing follow-up data were excluded from this analysis, and the remaining patients in the TCGA-STAD dataset were classified into two cohorts according to the median expression values of these hub genes. Log-rank *P*<0.05 was considered statistically significant.

### The correlation network and the relations of hub genes with clinical data

The correlation network of these hub genes was built using the igraph package in R software. The criterion of filtering was a cutoff >0.10. We also explored the relationship of the hub genes with clinical information (including tumor grade, individual cancer stages and nodal metastasis status) among STAD patients based on the UALCAN database (http://ualcan.path.uab.edu/) [19].

### GSEA of survival-related hub genes

GSEA is a computing approach that can recognize if the priorly defined gene set has statistical significance and concordant differences between two biological states [20]. We stratified STAD patients into two groups in light of the median expression values of survival-related hub genes. Afterward, the effects of their expression on many gene sets were explored to detect related KEGG pathways through the molecular signatures database (MSigDB) (c2.cp.kegg.all.v7.1.symbols.gmt) [21]. The permutation of every analysis was one thousand times. The selection criteria were as follows: |Normalized enrichment score (NES)| > 1, nominal (NOM) *P*-value <0.05 and FDR q-value <0.25.

### TIC abundance profile

We adopted the CIBERSORT computational approach to estimate the TIC abundance profiles among all STAD samples, which was followed by quality filtering [22]. Hence, 149 STAD samples with *P*<0.05 were included for the TIC profile in STAD samples and correlation analysis of 22 kinds of immune cells. We still explored the correlation of TIC proportion with the expression level of serpin family E member 1 (SERPINE1) in STAD.

## Results

### Selection of pivotal co-expression modules using WGCNA

To detect the functional module in STAD, two gene co-expression networks were established through the WGCNA package based on the GSE54129 and TCGA-STAD datasets. We detected eight modules in the GSE54129 dataset (Figure 2C) and 14 modules in the TCGA-STAD dataset (Figure 3C). Afterward, two heatmaps screened the relation between these modules and clinical traits (normal and STAD) in the GSE54129 (Figure 2D) and TCGA-STAD datasets (Figure 3D). The black module of GSE54129 and the turquoise module of the TCGA-STAD dataset were positively associated with normal tissues (black module in GSE54129: r = 0.85, *P*=7e-38, and turquoise module in the TCGA-STAD dataset: r = 0.34, *P*=1e-12).

### Selection of differentially co-expressed genes

**Figure 4:**
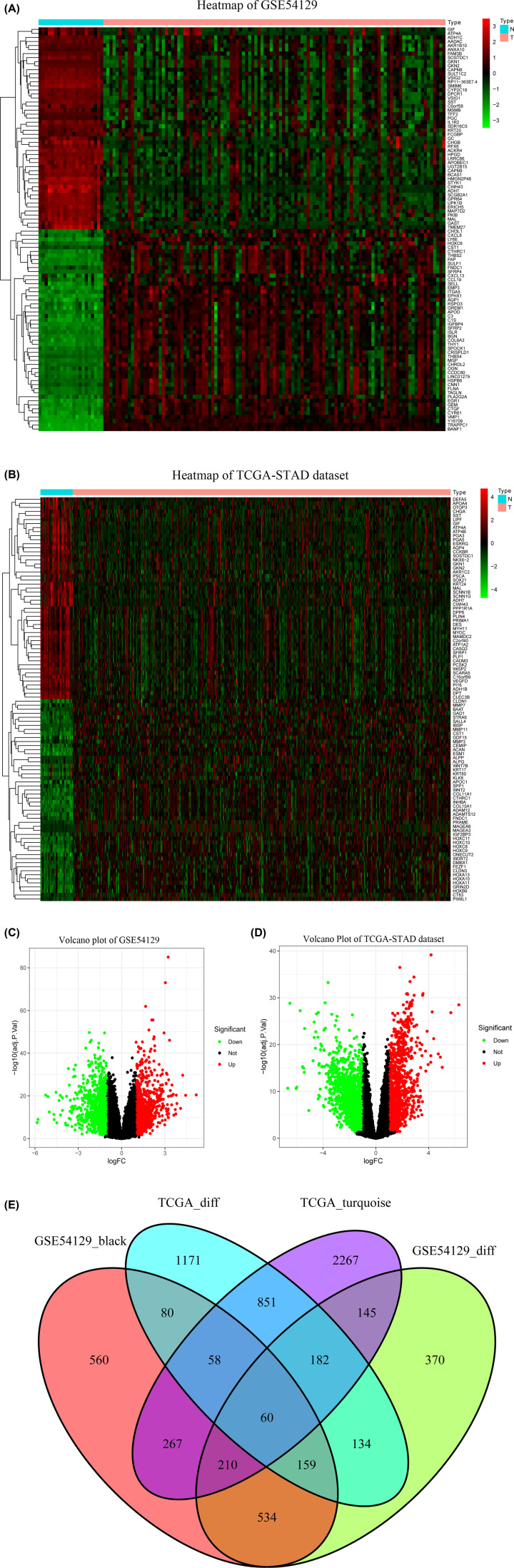
Identification of DEGs among GSE54129 and TCGA-STAD dataset with the cut-off criteria of |logFC| > 1 and adj.*P*<0.05 (**A**) Heatmap of top 50 up-regulated and 50 down-regulated DEGs of GSE54129. (**B**) Heatmap of top 50 up-regulated and 50 down-regulated DEGs of TCGA-STAD dataset. (**C**) Volcano plot of DEGs in GSE54129. (**D**) Volcano plot of DEGs in the TCGA-STAD dataset. (**E**) The Venn diagram of genes among the two DEG lists and the two lists of co-expression genes. A total of 60 overlapping differential co-expression genes are detected.

The two heatmaps showed the expression levels of 50 up-regulated and 50 down-regulated genes in the GSE54129 (Figure 4A) and TCGA-STAD datasets (Figure 4B). The volcano plots displayed that 1793 DEGs in the GSE54129 dataset (Figure 4C) and 2694 DEGs in the TCGA-STAD dataset (Figure 4D) were conspicuously dysregulated between STAD and non-tumorous tissues. Figure 4E clearly revealed that the intersection of two lists of DEGs (Supplementary Tables S1 and S2) and two lists of co-expressed genes (Supplementary Tables S3 and S4) contained a list of 60 genes (Supplementary Table S5), which were used for the next analysis.

### Functional enrichment analyses

**Figure 5:**
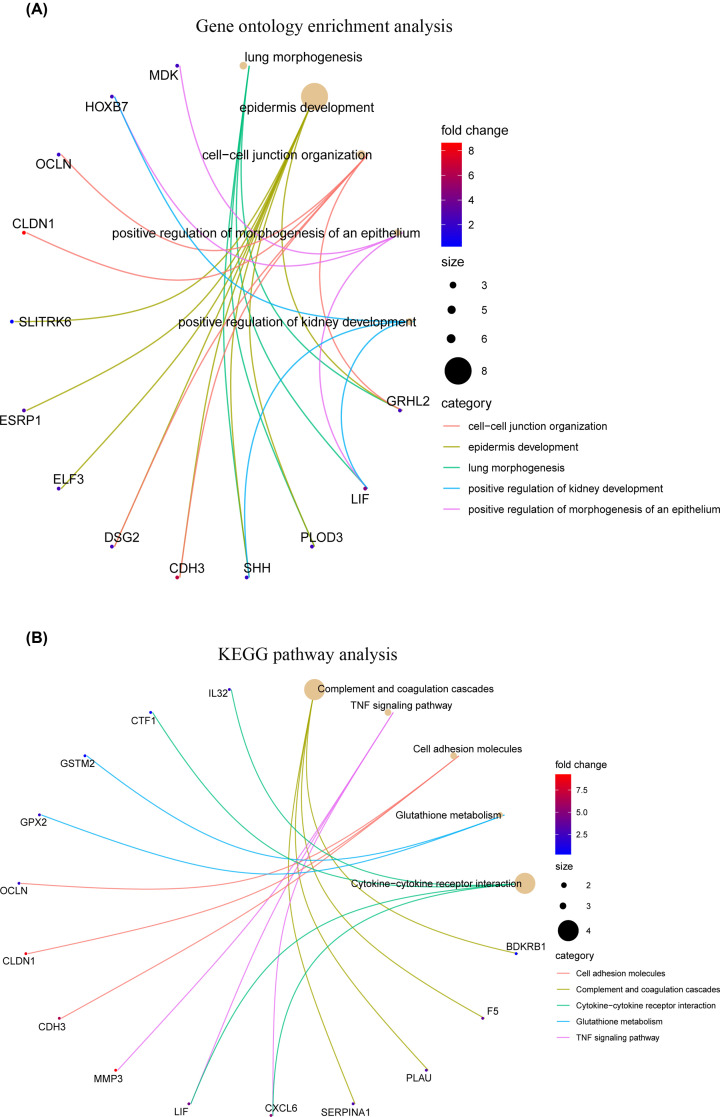
Functional enrichment analysis of differential co-expression genes using the clusterProfiler package (**A**) GO enrichment analysis of differential co-expression genes. (**B**) KEGG pathway analysis of differential co-expression genes.

To obtain further insights into the candidate biological functions, we conducted GO and KEGG pathway analyses of the 60 differentially co-expressed genes. According to the results of GO analysis, cell–cell junction organization, positive regulation of morphogenesis of the epithelium and epidermis development were significantly enriched (Figure 5A). In addition, KEGG pathway analysis of the 60 differentially co-expressed genes demonstrated that complement and coagulation cascades, cell adhesion molecules and cytokine–cytokine receptor interactions were mainly enriched (Figure 5B).

### PPI network construction and hub gene identification

**Figure 6:**
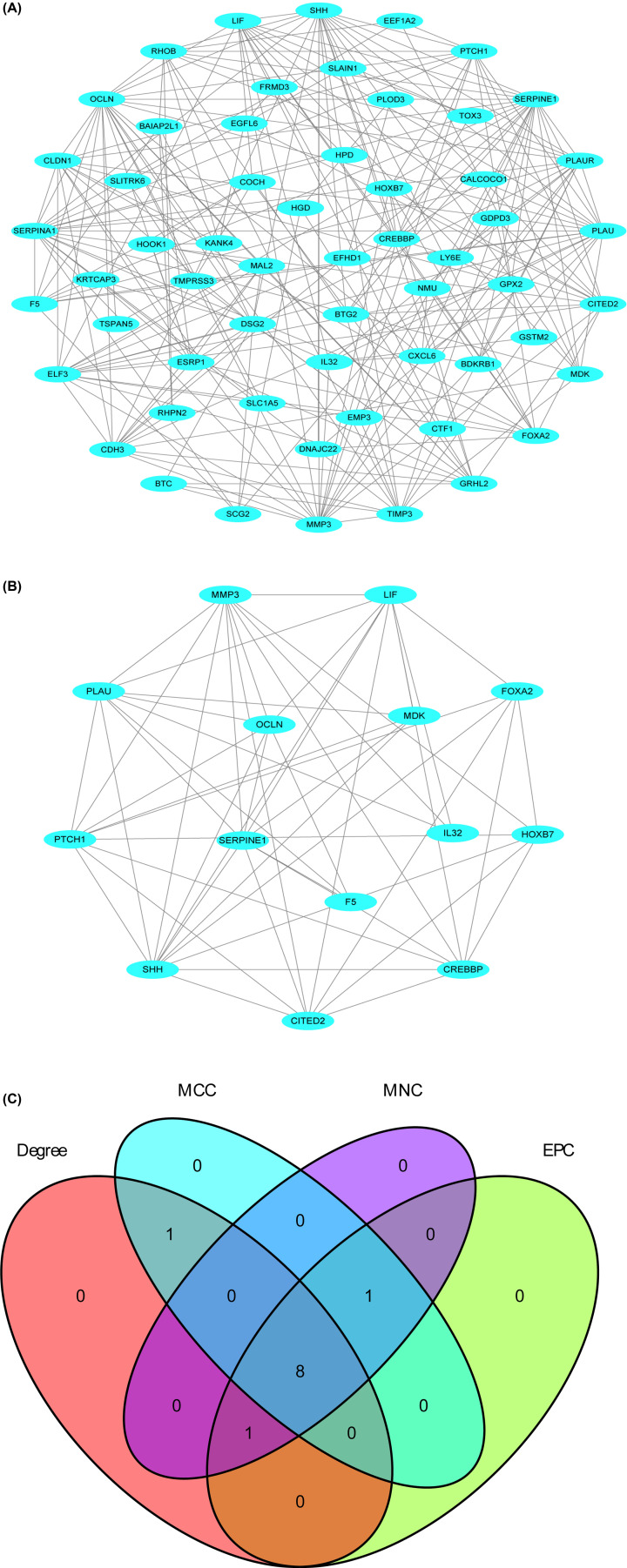
Visualization of the PPI network, the most significant modules and hub genes (**A**) PPI network of differential co-expression genes. (**B**) The most significant module from the PPI network. (**C**) The intersection of genes using the degree, EPC, MCC and MNC algorithms.

The PPI network of the 60 differentially co-expressed genes with 58 nodes and 227 edges was vividly illustrated (Figure 6A). Using the MCODE plugin, we found the most significant module of this PPI network, which contained 14 nodes and 52 edges (Figure 6B). Subsequently, the degree, EPC, MCC and MNC scores of the top 10 nodes were calculated through the CytoHubba plugin, and we took the intersection of results of the four algorithms (Figure 6C) to improve the reliability of hub genes. A total of eight genes (SERPINE1, OCLN, SHH, SERPINA1, PLAU, MMP3, ELF3 and PLAUR) were considered hub genes (Table 1).

**Table 1:** The selection of hub genes using the CytoHubba plugin

Gene symbol	Degree	EPC	MCC	MNC
SERPINE1	21	15.006	9592	21
OCLN	21	15.073	10137	20
SHH	20	15.106	8174	20
SERPINA1	19	14.439	5780	19
PLAU	18	14.466	9698	18
MMP3	18	14.607	8954	18
ELF3	16	13.456	1840	16
PLAUR	15	12.97	7477	14

### Survival analysis of hub genes

**Figure 7:**
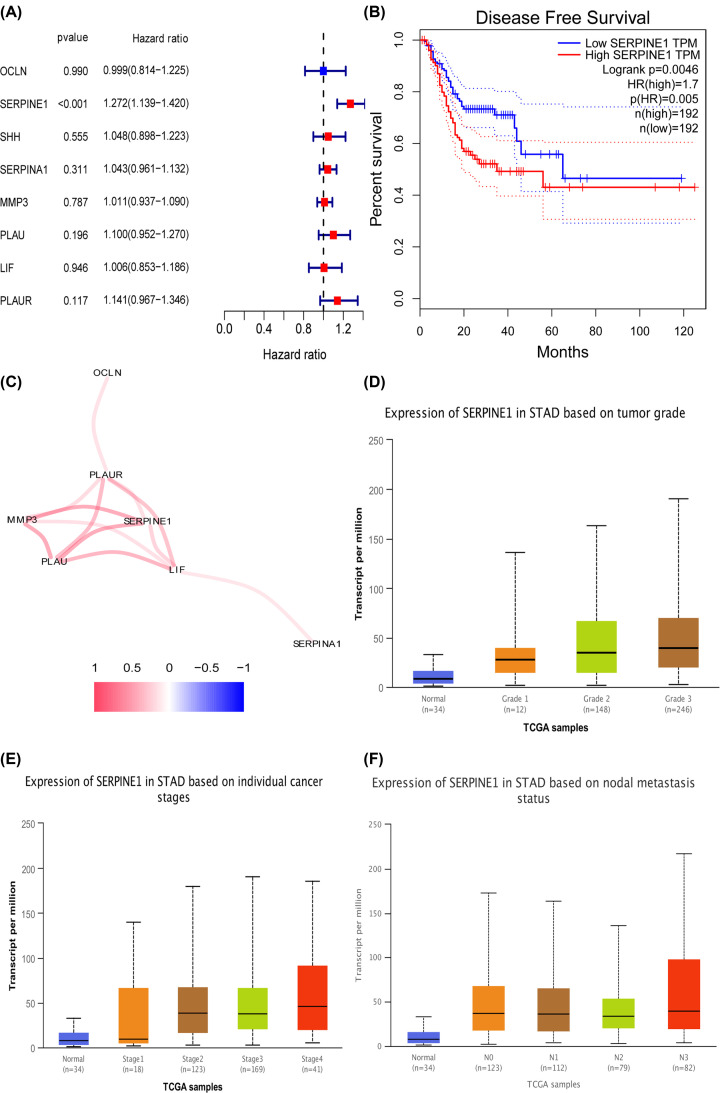
Survival analysis and relations of hub genes with clinical data (**A**) Univariate Cox regression analysis of OS of hub genes in GC. (**B**) The DFS analysis of SERPINE1in GC using the GEPIA database. (**C**) The correlation network of these hub genes. The correlation coefficients are represented by different colors. (**D**) The relation of SERPINE1 with tumor grades in GC. (**E**) The relation of SERPINE1 with individual cancer stages in GC. (**F**) The relation of SERPINE1 with nodal metastasis status in GC.

To explore the prognostic values of the eight hub genes in STAD, we conducted univariate Cox regression analysis of OS using the TCGA-STAD dataset. The higher expression of SERPINE1 was observed to be closely associated with shorter OS time among patients with STAD (hazard ratio (HR) = 1.27, 95% confidence interval (CI): 1.139–1.420, *P*=3.07E-05, Figure 7A). Moreover, the higher expression of SERPINE1 was significantly correlated with worse DFS among STAD patients (HR = 1.70, *P*=0.0046) (Figure 7B) using the GEPIA database.

### The correlation network and the relationship of hub genes with clinical data

The correlation network of these hub genes is presented in Figure 7C through the igraph package, and the shade of the color represents the magnitude of the correlation coefficients. Furthermore, we observed that the overexpression of SERPINE1 was highly correlated with poorer tumor grades (Figure 7D), higher individual cancer stages (Figure 7E), and more serious nodal metastasis status (Figure 7F) among patients with STAD.

### GSEA of survival-related hub genes

**Figure 8:**
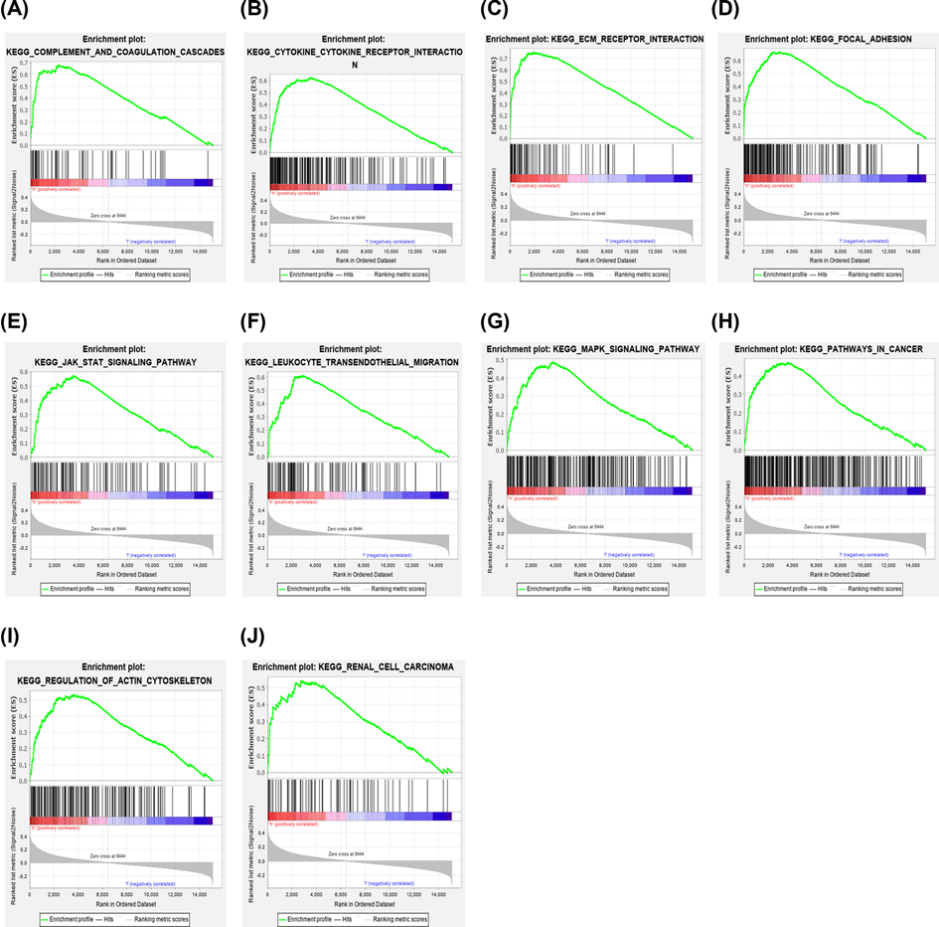
Enrichment plots by GSEA (**A**–**J**) Relative pathways associated with the expression of SERPINE1 are shown.

According to the outcomes of GSEA, we found that focal adhesion, extracellular matrix (ECM) receptor interaction, leukocyte transendothelial migration, regulation of actin cytoskeleton, MAPK signaling pathway, and pathways in cancers were highly associated with the expression of SERPINE1 (Figure 8A-J). In detail, the outcomes of GSEA of SERPINE1 in STAD are represented in Table 2 using the TCGA-STAD dataset.

**Table 2:** Relative pathways associated with the expression of SERPINE1 using GSEA

Gene	Name	ES	NES	NOM *P*-value	FDR q-value
SERPINE1	KEGG_FOCAL_ADHESION	0.66	2.38	<0.0001	0.001
	KEGG_ECM_RECEPTOR_INTERACTION	0.75	2.32	<0.0001	0.001
	KEGG_LEUKOCYTE_TRANSENDOTHELIAL_MIGRATION	0.61	2.27	<0.0001	0.001
	KEGG_CYTOKINE_CYTOKINE_RECEPTOR_INTERACTION	0.62	2.21	<0.0001	0.001
	KEGG_REGULATION_OF_ACTIN_CYTOSKELETON	0.53	2.15	<0.0001	0.001
	KEGG_MAPK_SIGNALING_PATHWAY	0.48	2.15	<0.0001	0.001
	KEGG_PATHWAYS_IN_CANCER	0.47	2.13	<0.0001	0.001
	KEGG_COMPLEMENT_AND_COAGULATION_CASCADES	0.68	2.10	<0.0001	0.001
	KEGG_JAK_STAT_SIGNALING_PATHWAY	0.57	2.08	<0.0001	0.002
	KEGG_RENAL_CELL_CARCINOMA	0.54	2.06	<0.0001	0.002

### Correlation of SERPINE1 with the proportion of TICs

**Figure 9:**
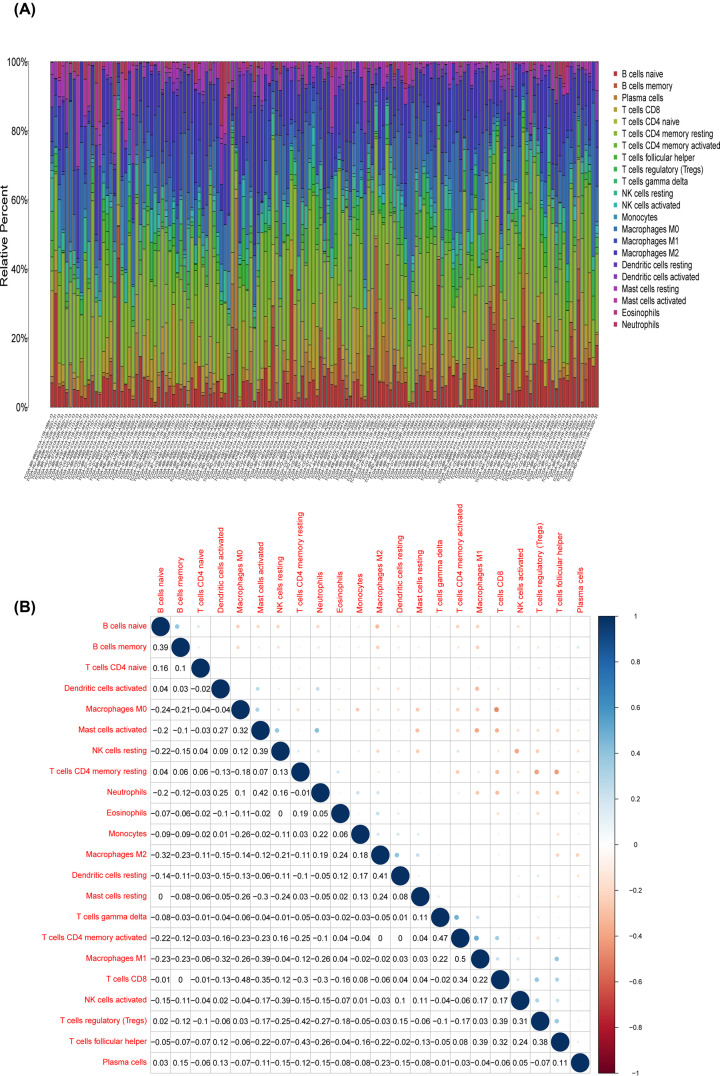
TIC profiles in GC samples and correlation analysis (**A**) The barplot showing the proportion of 22 kinds of TICs in GC samples. Column names of plot are sample ID. (**B**) Heatmap showing the correlation between 22 kinds of TICs and numeric in each tiny box suggesting the *P*-value of correlation between two kinds of cells. The shade of each tiny color box represents the corresponding correlation value between two cells, and the Pearson coefficient is applied in the significance test.

**Figure 10:**
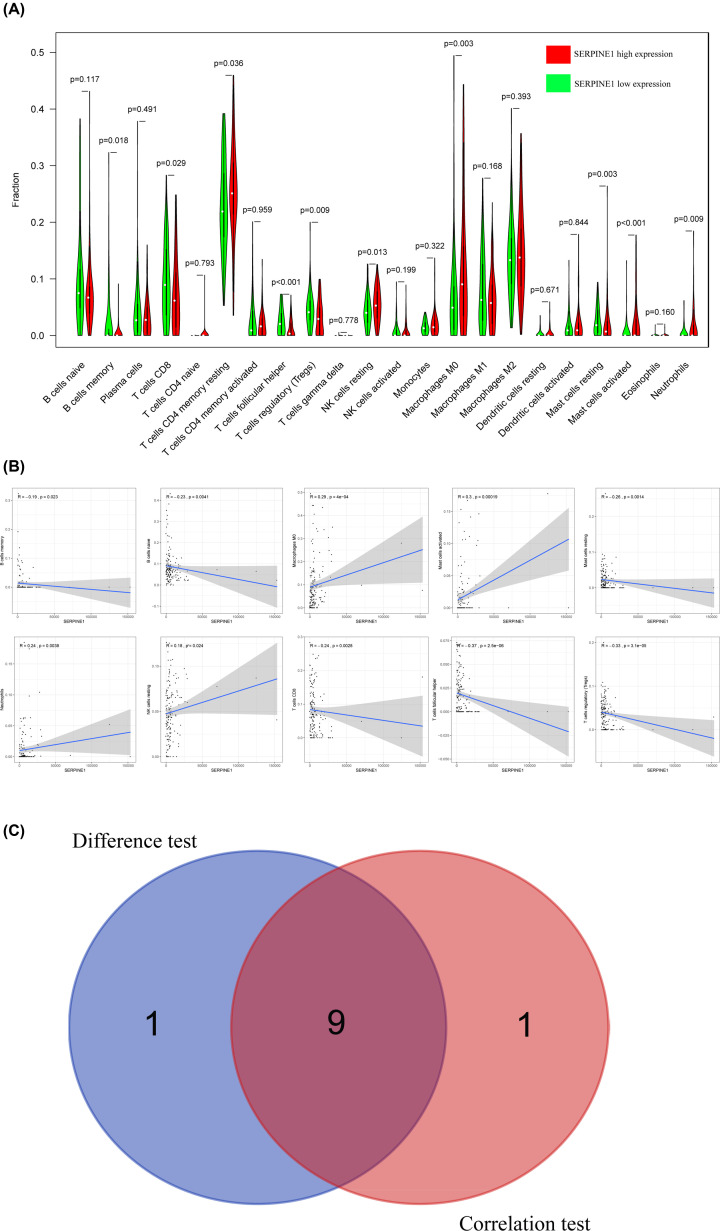
Correlation of TICs proportion with the expression of SERPINE1 (**A**) The Violin plot reveals the ratio differentiation of 22 kinds of immune cells between GC samples with low or high SERPINE1 expression relative to the median of SERPINE1 expression level, and the Wilcoxon rank-sum is applied for the significance test. (**B**) The Scatter plot displays the correlation of ten kinds of TICs proportion with the SERPINE1 expression (*P*<0.05). The red line in each plot is fitted linear model manifesting the proportion tropism of the immune cell along with the expression of SERPINE1, and the Pearson coefficient is applied for the correlation test. (**C**) Venn plot illustrates nine kinds of TICs correlated with SERPINE1 expression codetermined by difference and correlation tests displayed in violin and scatter plots, respectively.

To explore the relationship of SERPINE1 expression with the immune microenvironment, we analyzed the proportion of tumor-infiltrating immune subsets based on CIBERSORT. Twenty-two types of TICs in STAD samples are illustrated in Figure 9. The difference analysis showed ten types of TICs associated with SERPINE1 expression (Figure 10A), while the correlation analyses revealed ten kinds of TICs associated with SERPINE1 expression (Figure 10B). The intersection of the outcomes of difference and correlation analyses demonstrated that nine kinds of TICs were associated with the expression of SERPINE1 (Figure 10C). Namely, five types of TICs (memory B cells, CD8^+^ T cells, follicular helper T cells, regulatory T cells (Tregs) and resting mast cells) were negatively associated with SERPINE1 expression, while four types of TICs (M0 macrophages, activated mast cells, neutrophils and resting NK cells) were positively associated with SERPINE1 expression (Supplementary Table S6), suggesting that SERPINE1 might inhibit the immune-dominant status of the tumor microenvironment (TME).

## Discussion

As an aggressive solid-tumor malignancy with high incidence, GC has caused huge socioeconomic pressure on GC patients and their families. Currently, progression in the early diagnosis and prediction of the prognosis of GC remains limited. Hence, it is essential to detect tumor-specific indicators to monitor progression and predict the prognosis of GC patients. In our analysis, 60 differentially co-expressed genes were selected through integrated bioinformatics analysis based on the GSE54129 and TCGA-STAD datasets. Functional annotation analysis illustrated that cell–cell junction organization, positive regulation of morphogenesis of an epithelium, and cell adhesion molecules were primarily enriched. The intersection of genes using the degree, EPC, MCC and MNC algorithms were regarded as hub genes that were closely correlated with GC. Afterward, we found that SERPINE1 was highly correlated with the survival of patients with GC, and SERPINE1 expression was correlated with tumor grades, individual stages and N stages among GC patients. GSEA revealed that ECM receptor interaction, leukocyte transendothelial migration and pathways in cancers were associated with SERPINE1 expression. Finally, we found that the expression pattern of SERPINE1 influenced the immune activity of the TME in GC.

As a serine protease inhibitor, SERPINE1 can inhibit urokinase-type plasminogen (uPA) and tissue-type plasminogen activator (tPA) [22]. The plasminogen activator inhibitor-1 (PAI-1) encoded by SERPINE1 was an important regulator of the uPA system, which was supposed to inhibit the activation of uPA, but paradoxical studies revealed that SERPINE1 might play a role in promoting the carcinogenesis of many cancers [23]. Many investigations have implied that SERPINE1 is overexpressed in various cancers, including GC [24], head and neck cancer [25] and colorectal cancer [26], and this overexpression correlated with the aggressiveness and invasiveness of tumor cells. Additionally, the down-regulation of SERPINE1 suggested a suppressive effect on the phenotype of glioma tumor cells by activating the p53 signaling pathway and suppressing the adverse invasion and metastasis of nasopharyngeal carcinoma cells *in vitro* [27–28]. Xu et al. suggested that the overexpression of SERPINE1 was associated with poor prognosis in GC, and SERPINE1 was closely correlated with epithelial-to-mesenchymal transition (EMT) using qRT-PCR methods [29]. Similarly, Yang et al. indicated that SERPINE1 was apparently up-regulated in GC tissues, high expression of SERPINE1 was correlated with worse survival among patients with GC, and low expression of SERPINE1 had an inhibitory effect on the EMT process, while its overexpression had the reverse results [30]. In addition, based on the bioinformatics analysis of 293 GC and 196 normal tissues, Liao et al. found that SERPINE1 was remarkably overexpressed in GC tissues, and the overexpression of SERPINE1 was correlated with worse survival in GC patients [31]. SERPINE1 was reported to be up-regulated in STAD tissues compared with normal tissues, and the overexpression of SERPINE1 was closely associated with worse prognosis among STAD patients [32]. In addition, SERPINE1 was reported to have excellent prognostic value among patients with lower grade glioma [33]. SERPINE1 was observed to be up-regulated in glioma tissues using immunohistochemistry, and high expression of SERPINE1 was associated with reduced DFS and OS in glioma patients [34]. Considering these studies and our findings, we could conclude that SERPINE1 may closely participate in the carcinogenesis and development of GC, and SERPINE1 might act as an important biomarker of diagnosis, monitoring progression and predicting prognosis in GC patients.

Though some studies [29,30,35] have been published previously, our study has some correspondimg advantages compared with the similar studies. (1) Xu B et al. [29] just used the GEO database for the differential gene expression analysis, and another important database (TCGA) didn't be used. Yang JD et al. [30] just used the TCGA database for analysis, and the GEO database didn't be used. But we applied GEO and TCGA databases to find co-expressed differential genes instead of just one database, which may increase the credibility of our study. (2) Three of them [29,30,35] just performed differential gene expression analysis, and WGCNA didn't be carried out. But WGCNA and differential gene expression analysis were carried out in our study at the same time, which may enhance the reliability of our findings. (3) They didn't explore the correlation of SERPINE1 and tumor-infiltrating immune subsets in GC, but our study performed this, which increases novel content compared with them [29,30,35].

Admittedly, there were some limitations in our study. (1) though the GSE50129 and TCGA-STAD datasets had multiple samples of GC and nontumorous tissues, only the two datasets were used and analyzed in our analysis. (2) Although we used integrated bioinformatics methods to detect candidate prognostic biomarkers in GC, it might not be very precise for patients with different GC grades and stages. (3) We did not validate our findings by performing experiments, which would be an important issue that deserves further research in the future.

## Conclusion

This analysis was conducted to detect hub genes that may be correlated to the carcinogenesis and progression of GC using differential gene expression analysis and WGCNA. Eight hub genes were identified through the degree, EPC, MCC and MNC algorithms, and SERPINE1 was closely associated with the prognosis of GC patients. Furthermore, SERPINE1 may be a potential therapeutic and prognostic indicator in GC patients. Nonetheless, more studies are required to further validate and explore the biological relations among these differentially co-expressed genes in GC.

## Supplementary Material

Supplementary Tables S1-S6

## Data Availability

The datasets downloaded and analyzed during the current study are available on the TCGA and GEO databases: TCGA-STAD database: https://portal.gdc.cancer.gov/; GSE54129: https://www.ncbi.nlm.nih.gov/geo/query/acc.cgi?acc=GSE54129.

## Abbreviations

DEG: differentially expressed gene
DFS: disease-free survival
ECM: extracellular matrix
EMT: epithelial-to-mesenchymal transition
EPC: edge percolated component
FDR: false discovery rate
GC: gastric cancer
GEO: Gene Expression Omnibus
GEPIA: Gene Expression Profiling Interactive Analysis
GO: gene ontology
GSEA: Gene Set Enrichment Analysis
HR: hazard ratio
KEGG: Kyoto Encyclopedia of Genes and Genomes
MCC: maximal clique centrality
MCODE: molecular complex detection
MNC: maximum neighborhood component
OS: overall survival
PPI: protein–protein interaction network
SERPINE1: serpin family E member 1
STAD: stomach adenocarcinoma
TCGA: The Cancer Genome Atlas
TIC: tumor-infiltrating immune cell
TME: tumor microenvironment
TOM: topological overlap matrix
uPA: urokinase-type plasminogen
WGCNA: Weighted Gene Co-expression Network Analysis

## References

[1] Siegel R.L., Miller K.D. and Jemal A. (2020) Cancer statistics, 2020. CA Cancer J. Clin. 70, 7–30 10.3322/caac.21590 31912902

[2] Shi X.J., Wei Y. and Ji B. (2020) Systems biology of gastric cancer: perspectives on the omics-based diagnosis and treatment. Front. Mol. Biosci. 7, 203 10.3389/fmolb.2020.00203 33005629 PMC7479200

[3] Parisi A., Porzio G. and Ficorella C. (2020) Multimodality treatment in metastatic gastric cancer: from past to next future. Cancers (Basel) 12, E2598 10.3390/cancers12092598 32932914 PMC7563615

[4] Guerrini G.P., Esposito G., Magistri P. et al. (2020) Robotic versus laparoscopic gastrectomy for gastric cancer: The largest meta-analysis. Int. J. Surg. 82, 210–228 10.1016/j.ijsu.2020.07.053 32800976

[5] Arora I. and Tollefsbol T.O. (2020) Computational methods and next-generation sequencing approaches to analyze epigenetics data: profiling of methods and applications. Methods 187, 92–103 10.1016/j.ymeth.2020.09.008 32941995 PMC7914156

[6] Han Y., Wang W., Jia J. et al. (2020) WGCNA analysis of the subcutaneous fat transcriptome in a novel tree shrew model. Exp. Biol. Med. (Maywood) 245, 945–955 10.1177/1535370220915180 32216464 PMC7427174

[7] Nangraj A.S., Selvaraj G., Kaliamurthi S. et al. (2020) Integrated PPI- and WGCNA-retrieval of hub gene signatures shared between Barrett’s esophagus and esophageal adenocarcinoma. Front. Pharmacol. 11, 881 10.3389/fphar.2020.00881 32903837 PMC7438937

[8] Reddy R.R.S. and Ramanujam M.V. (2018) High throughput sequencing-based approaches for gene expression analysis. Methods Mol. Biol. 1783, 299–323 10.1007/978-1-4939-7834-2_15 29767369

[9] Robinson M.D., McCarthy D.J. and Smyth G.K. (2010) edgeR: a Bioconductor package for differential expression analysis of digital gene expression data. Bioinformatics 26, 139–140 10.1093/bioinformatics/btp616 19910308 PMC2796818

[10] Ritchie M.E., Phipson B., Wu D. et al. (2015) limma powers differential expression analyses for RNA-sequencing and microarray studies. Nucleic Acids Res. 43, e47 10.1093/nar/gkv007 25605792 PMC4402510

[11] Yu G., Wang L.G., Han Y. and He Q.Y. (2012) clusterProfiler: an R package for comparing biological themes among gene clusters. OMICS 16, 284–287 10.1089/omi.2011.0118 22455463 PMC3339379

[12] Ashburner M., Ball C.A., Blake J.A. et al. (2000) Gene ontology: tool for the unification of biology. The Gene Ontology Consortium. Nat. Genet. 25, 25–29 10.1038/75556 10802651 PMC3037419

[13] Kanehisa M., Furumichi M., Tanabe M., Sato Y. and Morishima K. (2017) KEGG: new perspectives on genomes, pathways, diseases and drugs. Nucleic Acids Res. 45, D353–D361 10.1093/nar/gkw1092 27899662 PMC5210567

[14] Franceschini A., Szklarczyk D., Frankild S. et al. (2013) STRING v9.1: Protein-protein interaction networks, with increased coverage and integration. Nucleic Acids Res. 41, D808–D815 10.1093/nar/gks1094 23203871 PMC3531103

[15] Smoot M.E., Ono K., Ruscheinski J., Wang P.L. and Ideker T. (2011) Cytoscape 2.8: New features for data integration and network visualization. Bioinformatics 27, 431–432 10.1093/bioinformatics/btq675 21149340 PMC3031041

[16] Bandettini W.P., Kellman P., Mancini C. et al. (2012) MultiContrast Delayed Enhancement (MCODE) improves detection of subendocardial myocardial infarction by late gadolinium enhancement cardiovascular magnetic resonance: a clinical validation study. J. Cardiovasc. Magn. Reson. 14, 83 10.1186/1532-429X-14-83 23199362 PMC3552709

[17] Chin C.H., Chen S.H., Wu H.H. et al. (2014) cytoHubba: identifying hub objects and sub-networks from complex interactome. BMC Syst. Biol. 8, S11 10.1186/1752-0509-8-S4-S11 25521941 PMC4290687

[18] Tang Z., Li C., Kang B. et al. (2017) GEPIA: a web server for cancer and normal gene expression profiling and interactive analyses. Nucleic Acids Res. 45, W98–W102 10.1093/nar/gkx247 28407145 PMC5570223

[19] Chandrashekar D.S., Bashel B., Balasubramanya S.A.H. et al. (2017) UALCAN: a portal for facilitating tumor subgroup gene expression and survival analyses. Neoplasia 19, 649–658 10.1016/j.neo.2017.05.002 28732212 PMC5516091

[20] Subramanian A., Tamayo P., Mootha V.K. et al. (2005) Gene set enrichment analysis: a knowledge-based approach for interpreting genome-wide expression profiles. Proc. Natl. Acad. Sci. U.S.A. 102, 15545–15550 10.1073/pnas.0506580102 16199517 PMC1239896

[21] Liberzon A., Subramanian A., Pinchback R. et al. (2011) Molecular signatures database (MSigDB) 3.0. Bioinformatics 27, 1739–1740 10.1093/bioinformatics/btr260 21546393 PMC3106198

[22] Chen B., Khodadoust M.S., Liu C.L., Newman A.M. and Alizadeh A.A. (2018) Profiling tumor infiltrating immune cells with CIBERSORT. Methods Mol. Biol. 1711, 243–259 10.1007/978-1-4939-7493-1_12 29344893 PMC5895181

[23] Li S., Wei X., He J. et al. (2018) Plasminogen activator inhibitor-1 in cancer research. Biomed. Pharmacother. 105, 83–94 10.1016/j.biopha.2018.05.119 29852393

[24] Zhang X., Zheng P., Li Z., Gao S. and Liu G. (2020) The somatic mutation landscape and RNA prognostic markers in stomach adenocarcinoma. Onco Targets Ther. 13, 7735–7746 10.2147/OTT.S263733 32801780 PMC7414981

[25] Arroyo-Solera I., Pavón M.Á., León X. et al. (2019) Effect of serpinE1 overexpression on the primary tumor and lymph node, and lung metastases in head and neck squamous cell carcinoma. Head Neck 41, 429–439 30548470 10.1002/hed.25437

[26] Liu Y., Li C., Dong L., Chen X. and Fan R. (2020) Identification and verification of three key genes associated with survival and prognosis of COAD patients via integrated bioinformatics analysis. Biosci. Rep. 40, BSR20200141 10.1042/BSR20200141 32936304 PMC7522359

[27] Wu D.M., Wang S., Wen X. et al. (2018) MircoRNA-1275 promotes proliferation, invasion and migration of glioma cells via SERPINE1. J. Cell. Mol. Med. 22, 4963–4974 10.1111/jcmm.13760 30024092 PMC6156288

[28] Sang Y., Chen M.Y., Luo D. et al. (2015) TEL2 suppresses metastasis by down-regulating SERPINE1 in nasopharyngeal carcinoma. Oncotarget 6, 29240–29253 10.18632/oncotarget.5074 26335051 PMC4745723

[29] Xu B., Bai Z., Yin J. and Zhang Z. (2019) Global transcriptomic analysis identifies SERPINE1 as a prognostic biomarker associated with epithelial-to-mesenchymal transition in gastric cancer. PeerJ 7, e7091 10.7717/peerj.7091 31218131 PMC6563800

[30] Yang J.D., Ma L. and Zhu Z. (2019) SERPINE1 as a cancer-promoting gene in gastric adenocarcinoma: facilitates tumour cell proliferation, migration, and invasion by regulating EMT. J. Chemother. 31, 408–418 10.1080/1120009X.2019.1687996 31724495

[31] Liao P., Li W., Liu R. et al. (2018) Genome-scale analysis identifies SERPINE1 and SPARC as diagnostic and prognostic biomarkers in gastric cancer. Onco Targets Ther. 11, 6969–6980 10.2147/OTT.S173934 30410354 PMC6199229

[32] Luo S.S., Liao X.W. and Zhu X.D. (2019) Genome-wide analysis to identify a novel microRNA signature that predicts survival in patients with stomach adenocarcinoma. J. Cancer 10, 6298–6313 10.7150/jca.33250 31772663 PMC6856753

[33] Wang H., Wang X., Xu L., Zhang J. and Cao H. (2020) Prognostic significance of age related genes in patients with lower grade glioma. J. Cancer 11, 3986–3999 10.7150/jca.41123 32328202 PMC7171497

[34] Vachher M., Arora K., Burman A. and Kumar B. (2020) NAMPT, GRN, and SERPINE1 signature as predictor of disease progression and survival in gliomas. J. Cell. Biochem. 121, 3010–3023 10.1002/jcb.29560 31710121

[35] Li L., Zhu Z., Zhao Y. et al. (2019) FN1, SPARC, and SERPINE1 are highly expressed and significantly related to a poor prognosis of gastric adenocarcinoma revealed by microarray and bioinformatics. Sci. Rep. 9, 7827 10.1038/s41598-019-43924-x 31127138 PMC6534579

