# Supplementary material for: Identification of pivotal genes associated with the prognosis of gastric carcinoma through integrated analysis
Source: Biosci Rep. 2021 Apr 14;41(4):BSR20203676. doi: 10.1042/BSR20203676 (PMC8047542; doi:10.1042/BSR20203676)
Supplement: Supplementary Tables S1-S6 [file BSR-2020-3676_supp.zip › BSR-2020-3676_suppS6.docx]

Table S6 TICs co-determined by difference test and correlation test

| Cell | Correlation test（P-value） | Difference test（P-value） |
| --- | --- | --- |
| B cells memory | -0.19 (0.023) | 0.018 |
| T cells CD8 | -0.24 (0.003) | 0.029 |
| T cells follicular helper | -0.37 (<0.001) | <0.001 |
| T cells regulatory (Tregs) | -0.33 (0.000) | 0.009 |
| NK cells resting | 0.18 (0.024) | 0.013 |
| Macrophages M0 | 0.29 (<0.001) | 0.003 |
| Mast cells resting | -0.25 (0.001) | 0.003 |
| Mast cells activated | 0.30 (<0.001) | <0.001 |
| Neutrophils | 0.24 (0.004) | 0.009 |

**Abbreviations:** TICs: tumor-infiltrating immune cells.
